# Quality assurance of surgical interventions for pancreatic cancer: systematic review of multicentre randomized clinical trials

**DOI:** 10.1093/bjsopen/zraf082

**Published:** 2025-08-14

**Authors:** Jack A Helliwell, Sophie Rozwadowski, Jing Yi Kwan, Melissa Bautista, Shailesh V Shrikhande, Deborah D Stocken, Natalie S Blencowe, Andrew M Smith, Samir Pathak

**Affiliations:** Leeds Institute of Medical Research at St James’s, University of Leeds, Leeds, UK; Department of Pancreatic Surgery, Leeds Teaching Hospitals NHS Trust, Leeds, UK; Department of General Surgery, Barnsley Hospitals NHS Foundation Trust, Barnsley, UK; Department of Pancreatic Surgery, Leeds Teaching Hospitals NHS Trust, Leeds, UK; Department of Pancreatic Surgery, Leeds Teaching Hospitals NHS Trust, Leeds, UK; Department of Cancer Surgery, Tata Memorial Centre, Mumbai, India; Leeds Institute of Medical Research at St James’s, University of Leeds, Leeds, UK; Clinical Trials Research Unit, Leeds Institute of Clinical Trials Research, University of Leeds, Leeds, UK; Leeds Institute of Medical Research at St James’s, University of Leeds, Leeds, UK; Bristol Centre for Surgical Research, Bristol Medical School, Bristol, UK; Department of Pancreatic Surgery, Leeds Teaching Hospitals NHS Trust, Leeds, UK; Department of Pancreatic Surgery, Leeds Teaching Hospitals NHS Trust, Leeds, UK; Clinical Trials Research Unit, Leeds Institute of Clinical Trials Research, University of Leeds, Leeds, UK

## Abstract

**Background:**

Surgical interventions for pancreatic cancer are complex due to numerous interacting components. This complexity can make the design and conduct of randomized clinical trials (RCTs) challenging due to variations in how surgical interventions are delivered across centres and surgeons. Quality assurance (QA) methods, such as those described within the CONSORT recommendations for non-pharmacological interventions (CONSORT-NPT), attempt to mitigate this. The extent of the adoption of such QA methods in RCTs evaluating surgical interventions for pancreatic cancer is unclear.

**Methods:**

A systematic review was conducted on multicentre RCTs evaluating surgical interventions for pancreatic cancer. Data were extracted within four QA domains described within the CONSORT-NPT checklist: surgical intervention description, standardization, adherence, and clinician and unit expertise.

**Results:**

Of 2374 studies identified, 45 were eligible for inclusion in this review. Thirty-eight RCTs (84%) described the intervention and 20 (44%) attempted to standardize techniques. Information about permitted flexibility in surgical interventions was described in 14 RCTs (31%). Fourteen studies (31%) described methods used to measure adherence to the intervention, with intra-operative photographs/videos (ten studies) being the most common. Nineteen studies (42%) detailed surgeon or unit expertise, and six (13%) used credentialing criteria.

**Conclusion:**

Although most RCTs described the intervention, reporting on standardization, adherence, and expertise was often lacking. This may affect RCT results and compromise the extent to which observed differences in clinical outcomes are due to the actual intervention being delivered. More rigorous application and reporting of QA measures are needed to improve confidence in the results of future RCTs, which may, in turn, enhance implementation in clinical practice.

## Teaser

This systematic review evaluated the quality assurance (QA) methods in multicentre randomized clinical trials (RCTs) of surgical interventions for pancreatic cancer, focusing on the four QA domains outlined in the CONSORT recommendations for non-pharmacological interventions: intervention description, standardization, adherence, and clinician/unit expertise. Although most trials described the intervention, reporting on standardization, adherence, and expertise was often insufficient.

## Introduction

Surgical procedures for pancreatic cancer are considered to be complex interventions because they comprise multiple components that may act independently or interdependently to influence outcomes. Both pancreatoduodenectomy and left pancreatectomy are technically challenging and have been modified over time with the aim of improving outcomes for patients^1^.

The benchmark for evaluating such interventions is to undertake multicentre randomized clinical trials (RCTs). Although the use of multiple centres is an important characteristic of pragmatic trial design to improve generalizability, it potentially introduces heterogeneity in technical performance due to the fact that surgeons inherently undertake procedures in slightly different ways and have variable skill levels^2^. A lack of consideration for intervention standardization and surgeon expertise in the context of RCTs therefore has the potential to introduce bias and compromise the detection of differences in clinical outcomes between the techniques being evaluated^2^. This is because non-standardization may lead to partial homogenization of treatment arms^3^.

The need for methodological rigour to reduce such biases is acknowledged in reporting guidance, such as the CONSORT extension for non-pharmacological treatments (CONSORT-NPT)^4^. These guidelines encompass invasive surgical interventions, such as those used in pancreatic cancer surgery. CONSORT-NPT^4^ suggests that ‘precise details of experimental treatment’, ‘details on whether and how the interventions were standardized’, ‘details on whether and how adherence of care providers to the protocol was assessed’, and ‘information about the expertise of the care providers’ should all be described in trial reports. Collectively, these facets outlined in the CONSORT-NPT checklist serve as recommendations to optimize the quality assurance (QA) of the surgical interventions being evaluated^5^.

In light of these considerations, the primary aim of this systematic review was to identify and summarize approaches to QA within multicentre RCTs evaluating surgical interventions for pancreatic cancer.

## Methods

### Study design

A systematic review was performed according to a predefined protocol. The review was not registered in the PROSPERO database because it focused on an aspect of trials methodology and therefore did not meet the criteria for registration. The review was conducted according to the PRISMA guidelines^6^.

### Search strategy

A systematic search of MEDLINE (via Ovid), Embase (via Ovid), and Cochrane Central Registry of Controlled Trials (via Ovid) databases was performed for articles published between 1 January 2000 and 31 December 2024 to reflect contemporary practice (*Table S1*). Two independent investigators (J.A.H., S.R.) reviewed titles, abstracts, and full-text papers. Discrepancies were resolved through discussion with the senior investigator (S.P.).

### Eligibility criteria

Eligible studies adhered to a multicentre RCT design, defined as clinical investigations conducted at multiple sites involving participants randomly allocated to at least two study arms. The assessment focused exclusively on multicentre RCTs because these constitute the most robust evidence for surgical interventions and are susceptible to increased variability across a larger pool of surgeons and study locations. Furthermore, eligible studies were required to evaluate a surgical intervention, surgical technique modification, or variation in approach to pancreatic resection, specifically during pancreatoduodenectomy or left pancreatectomy. Studies evaluating radiologically guided or endoscopic interventions were excluded. Studies evaluating perioperative interventions, such as enhanced recovery after surgery, chemoradiotherapy, or pharmacological interventions were also excluded. The review included studies involving patients undergoing pancreatic resection for malignant indications, as well as those with mixed cohorts that included both malignant and benign pathology. Studies exclusively focused on benign disease were excluded, because the primary aim of the review was to examine surgical QA in the context of pancreatic cancer, where the complexity and high-risk nature of the disease heightens the importance of rigorous trial design and delivery. In addition, articles published in a language other than English were excluded.

### Data extraction and charting

Data were extracted from included studies by one of four investigators. A random 20% sample of extracted data was independently verified by the senior investigator (S.P.). This verification process involved re-extracting the data independently from the original extraction, followed by a comparison to identify and resolve any discrepancies. Data extraction was completed using a semistructured data extraction spreadsheet in Microsoft^®^ Excel (Microsoft, Redmond, WA, USA).

#### Surgical intervention description (CONSORT-NPT items 5 and 5a)

Reporting of details about the interventions was assessed by recording verbatim descriptions of the components and steps of the procedure (item 5a)^4^. A description was deemed to have been provided if anything more than the name of the intervention or device to be implanted was reported.

#### Surgical intervention standardization (CONSORT-NPT item 5b)

Standardization was defined as the process of making an intervention conform to a standard (that is, falling into an accepted range of quality)^4^. Reporting on the standardization of interventions was deemed to have been provided if studies included specific details about the criteria for using the intervention (or any of its components or steps) were reported. To aid the identification and assessment of standardization processes, trial reports were examined for the term ‘standardized’ or synonyms like ‘uniform’ or ‘mandatory’, as well as methods used to ensure consistency (for example, operative manual).

An additional data point was also included, noting whether there was any permitted flexibility with which the operative steps or components were delivered, or whether they needed to be rigidly followed (that is, whether they were mandatory or prohibited).

#### Intervention adherence (CONSORT-NPT item 5d)

Any reporting of adherence to the intervention (defined as the degree to which an intervention was conducted according to the protocol or as outlined by its designers) was recorded, including details of how this was measured^4^.

#### Clinician and unit expertise (CONSORT-NPT item 15)

Any provision of information about clinician qualification, grade, and the number of resections previously undertaken (including those using the new technique) was recorded^4^. Similar information was collected about any criteria required by units for trial eligibility.

### Statistical analysis

Data were analysed descriptively using proportions. No quantitative syntheses of outcomes or assessment of study quality was undertaken. A narrative synthesis of data collected from eligible studies is presented, taking into consideration whether the trial pertained to either pancreatoduodenectomy or left pancreatectomy.

## Results

### Characteristics of included studies

**Fig. 1:**
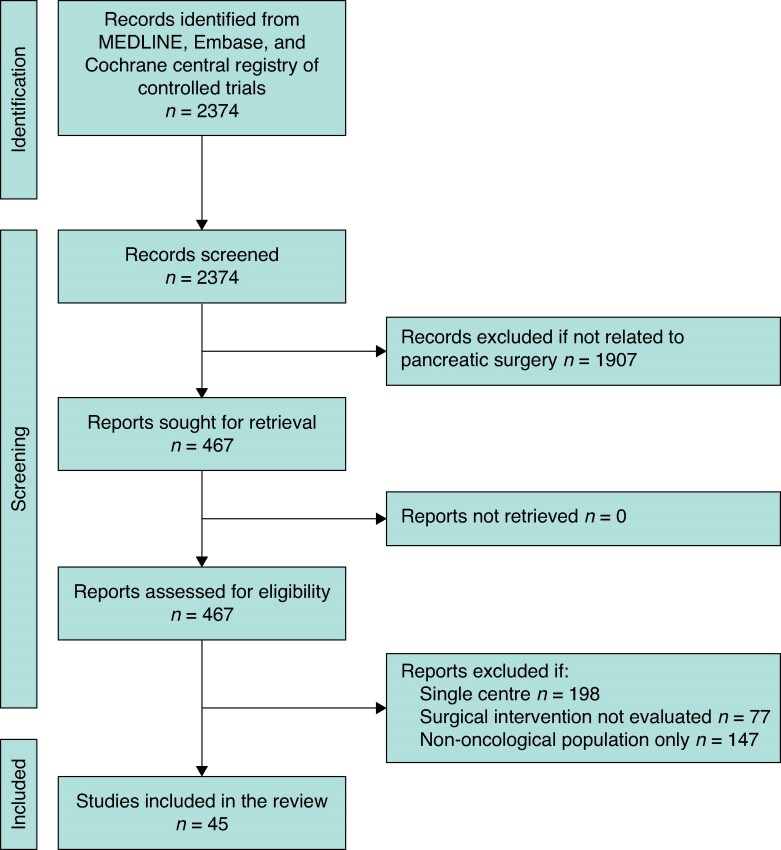
PRISMA flow diagram of study eligibility

Systematic searches identified 2374 studies in total, 45 of which were included in the review (*Fig. 1*). Eligible studies originated from a range of geographical locations, including Japan (9 studies), the US (8), Germany (5), China (4), and the Netherlands (3). Many were published in high-impact journals, including *Annals of Surgery* (16 studies), *BJS* (7), *JAMA Surgery* (2), and *The Lancet Gastroenterology and Hepatology* (2). The multicentre RCTs were performed in 2–45 centres, with sample sizes ranging from 73 to 656. Twenty-seven of the included studies evaluated surgical interventions during pancreatoduodenectomy (*Table S2*)^7–33^. Eighteen studies pertained to left pancreatectomy (*Table S3*)^34–51^. Various interventions were identified, including reconstruction techniques (11 studies), stump reinforcement (left pancreatectomy only; 11), the extent of surgical resection (7), sealants/stents (5), minimally invasive surgery (4), dissection approaches (2), transection methods (2), and drain placement (3).

### Surgical intervention description (CONSORT-NPT items 5 and 5a)

Of the 45 included studies, 38 (84%) provided a description of the surgical intervention (that is, more information than just the name of the procedure; *Table 1*). A range of descriptions for various interventions were noted (*Table S4*). The level of detail often varied depending on the complexity of the intervention being evaluated.

**Table 1:** Quality assurance measures reported in pancreatic surgery trials

Author (year)	Surgical intervention description	Surgical intervention standardization		Measurement of intervention adherence	Clinician and unit expertise	
		Intervention standardization	Details about flexibility		Surgeon entry criteria	Unit entry criteria
**Pancreatoduodenectomy**						
Wang *et al.* (2023)^7^	Yes	No	Yes	Yes	Yes	No
Lin *et al.* (2023)^8^	Yes	Yes	No	Yes	Yes	Yes
Yamada *et al.* (2020)^9^	Yes	Yes	No	Yes	No	No
Welsch *et al.* (2022)^10^	Yes	Yes	Yes	No	No	No
Toyama *et al.* (2021)^11^	Yes	Yes	Yes	Yes	No	Yes
Wang *et al.* (2021)^12^	Yes	No	No	Yes	Yes	No
Sabater *et al.* (2019)^13^	Yes	Yes	No	No	No	Yes
van Hilst *et al.* (2019)^14^	Yes	No	No	Yes	Yes	Yes
Schindl *et al.* (2018)^15^	Yes	Yes	Yes	No	No	Yes
Witzigmann *et al.* (2016)^16^	Yes	Yes	No	No	No	No
Jang *et al.* (2016)^17^	Yes	No	Yes	Yes	Yes	No
Sakamoto *et al.* (2016)^18^	Yes	Yes	No	No	No	No
Keck *et al.* (2016)^19^	No	No	No	No	No	Yes
Van Buren *et al.* (2014)^20^	No	No	Yes	No	No	Yes
Jang *et al.* (2014)^21^	Yes	No	Yes	Yes	Yes	No
Figueras *et al.* (2013)^22^	Yes	No	No	No	No	No
Topal *et al.* (2013)^23^	Yes	No	Yes	No	Yes	No
Ke *et al.* (2013)^24^	Yes	No	No	No	No	No
Uzunoglu *et al.* (2012)^25^	No	Yes	No	Yes	No	No
Nimura *et al.* (2012)^26^	Yes	Yes	No	Yes	Yes	No
Pessaux *et al.* (2011)^27^	Yes	No	No	No	No	No
Berger *et al.* (2009)^28^	Yes	No	No	No	No	No
Duffas *et al.* (2005)^29^	No	No	No	No	No	No
Tran *et al.* (2004)^30^	Yes	No	No	No	No	No
Suc *et al.* (2003)^31^	Yes	No	No	No	No	No
Tran *et al.* (2002)^32^	Yes	No	No	No	No	No
Takano *et al.* (2000)^33^	Yes	No	No	No	No	No
**Distal pancreatectomy**						
Korrel *et al.* (2023)^34^	Yes	Yes	Yes	No	Yes	Yes
Merdrignac *et al.* (2022)^35^	Yes	No	No	No	No	No
Uranues *et al.* (2021)^36^	Yes	No	No	No	No	No
Yamada *et al.* (2021)^37^	Yes	Yes	No	Yes	No	No
Landoni *et al.* (2022)^38^	Yes	No	No	No	Yes	No
Wennerblom *et al.* (2021)^39^	Yes	No	No	No	No	No
Kondo *et al.* (2019)^40^	Yes	No	No	No	No	No
de Rooij *et al.* (2019)^41^	Yes	Yes	Yes	No	Yes	Yes
Van Buren *et al.* (2017)^42^	No	No	No	Yes	No	Yes
Uemura *et al.* (2017)^43^	Yes	Yes	No	No	No	No
Jang *et al.* (2017)^44^	Yes	Yes	Yes	No	No	Yes
Shubert *et al.* (2016)^45^	Yes	Yes	Yes	No	No	No
Park *et al.* (2016)^46^	Yes	Yes	No	Yes	No	No
Kawai *et al.* (2016)^47^	Yes	No	No	No	No	No
Cunha *et al.* (2015)^48^	Yes	No	No	No	No	No
Carter *et al.* (2013)^49^	No	Yes	No	No	No	No
Montorsi *et al.* (2012)^50^	Yes	Yes	Yes	No	No	No
Diener *et al.* (2011)^51^	No	Yes	Yes	Yes	No	Yes

### Surgical intervention standardization (CONSORT-NPT item 5b)

Efforts to standardize technique during delivery of the intervention were described in 20 RCTs (44%) (*Table 1*). The level of standardization varied among these studies (*Table S5*). Some studies demonstrated a thorough and intentional effort to maintain standardization. In contrast, others mentioned standardization but the exact methods were less clearly defined. Information about permitted flexibility in surgical interventions was described in 14 RCTs (31%).

Additional information regarding aspects of an intervention explicitly labelled as prohibited was only present in two studies^18,40^.

### Intervention adherence (CONSORT-NPT item 5d)

Fourteen studies (31%) described methods used to monitor performance and adherence to the interventions during the trial (*Table S6*).

Various approaches were used, including intraoperative photographs (9 studies), intraoperative videos (1), surgeon self-declaration (2), pathological specimen review (1), a review of case report forms (1), and a review of operation notes (1)^7–9,11,12,14,17,21,25,26,37,42,46,51^.

### Clinician and unit expertise (CONSORT-NPT item 15)

Nineteen studies (42%) included details regarding the level of surgeon and/or unit experience within the trial (*Table S7*). Of these studies, 4 (9%) provided information on both surgeon and unit expertise. Seven studies (16%) described surgeon expertise alone, and 8 (19%) described unit expertise alone.

Six studies (13%) outlined credentialing methods based on prospectively defined entry criteria for surgeons and/or units. These criteria commonly involved establishing a minimum case number for the specific procedure under investigation. In one study, surgeons were also required to have completed a training program in laparoscopic left pancreatectomy and pancreatoduodenectomy to participate^14^.

## Discussion

This systematic review examined the reporting of four key domains of QA within multicentre RCTs evaluating surgical interventions for pancreatic cancer, with specific reference to the CONSORT-NPT checklist. The findings indicate that most studies provided at least some description of the intervention under evaluation. Reporting of intervention standardization, adherence, and surgeon/unit expertise were all reported less frequently. These shortcomings in QA potentially undermine the reliability and validity of findings from surgical trials and underscore the critical need for stricter adherence to these elements to ensure reliable and high-quality evidence in pancreatic surgery research.

Previous assessments of trial conduct within the field of surgery, and specifically in pancreatic surgery, have been conducted. A systematic review^52^ of surgical RCTs that had been published in 1999, 2009, and 2019 found that although the volume of published surgical RCTs worldwide remained stable over the past decade, their methodological quality had improved. A separate systematic review^53^ of RCTs in pancreatic surgery specifically evaluated both the quantity and quality of RCTs conducted over the last three decades, finding that the overall quality of RCTs, as measured using the Cochrane risk-of-bias tool, was moderate^53^. Notably, all domains of the Cochrane risk-of-bias tool, except blinding, demonstrated significant improvement over time. It is important to highlight that the Cochrane risk-of-bias tool assesses methodological quality but does not encompass the QA elements covered by the CONSORT-NPT checklist used in the present study^54^.

Although this study marks the first exploration of QA in pancreatic RCTs, similar reviews have been conducted in other clinical contexts. For example, a systematic review^5^ examined QA according to the same CONSORT-NPT standards in RCTs involving invasive procedures for assisted birth. Encouragingly, intervention description (84 *versus* 55%) was higher in pancreatic RCTs than in those involving assisted birth. The methods to monitor adherence to interventions were similarly low in the present review and in the systematic review^5^ of RCTs involving invasive procedures for assisted birth (31 *versus* 21%). The standardization of surgical interventions (44% *versus* 64%) and credentialing methods based on clinician and unit entry criteria (42% *versus* 64%) were less common in pancreatic RCTs than in RCTs in assisted vaginal birth^5^.

This limited adoption of specific QA domains, such as intervention standardization, monitoring intervention adherence, and credentialing surgeon/unit expertise, may be attributed to a combination of a lack of awareness regarding the significance of these components in clinical trial design and the use of imprecise language in existing frameworks. For example, in the case of CONSORT-NPT, there is ambiguity in the language used to define descriptions, standardization, and adherence^55–57^. Although CONSORT-NPT recommends providing precise details of the experimental treatment, it does not offer clear definition of what constitutes precision. In the present study, it was particularly challenging to distinguish between what constituted sufficient intervention description and what constituted intervention standardization. It is also important to recognize that the feasibility and degree of standardization required may differ depending on the trial type: whether explanatory or pragmatic. Explanatory trials, which assess the efficacy of interventions, often require detailed descriptions because the interventions are typically novel, and safety needs to be evaluated in a tightly controlled setting. In contrast, pragmatic trials, which focus on determining whether interventions are effective in real-world settings, are often multicentre studies involving larger populations. Achieving complete standardization across every procedural component in these trials is not only highly challenging but may also be unrealistic. Such an approach could fail to reflect the inherent variability of routine clinical practice. Instead, a balance may need to be struck between ensuring adequate standardization of key components and allowing flexibility in others. As alternative trial designs, such as stepped-wedge, registry-based, and trial-within-cohorts, become more widely used to increase pragmatism, it will be interesting to explore whether these designs help lower barriers to implementing QA measures^58^. Although further investigation is needed, it is plausible that stepped-wedge trials could make intervention standardization easier to achieve across clusters.

This study does have limitations. Because the sample of RCTs in this study was limited to those involving multiple centres, the findings may not generalizable. It is possible that these trials were more likely to incorporate QA measures than single-centre trials, potentially underestimating the extent of the issue. Data were collected only for the intervention group, using it as a proxy to reflect QA in the trial as a whole. It is important to highlight the need for QA measures across both intervention and control groups to ensure both are delivered as intended. Only protocols or related documents mentioned explicitly by authors in the trial reports were retrieved, which may have led to some omissions. Some trial registries, such as ClinicalTrials.gov, were not searched for additional protocol information, which may have provided further insights into QA domains. Although the review focused on surgical interventions for pancreatic cancer, studies with mixed cohorts, including malignant and benign disease, were included. This may have introduced additional variability in surgical QA across the included studies. It was also assumed that QA domains were not conducted if they were absent from the published report; some studies may have used these measures but not reported them in the manuscript.

This study provides important data on the utilization of methods of surgical QA within pancreatic surgery RCTs, and the QA was founded low. This may compromise the extent to which observed differences in clinical outcomes are due to the intervention being evaluated. Forthcoming pancreatic RCTs should prioritize the following actions during the design phase: consideration for which intervention components or steps should be mandatory and those that can be delivered flexibly (that is, non-mandatory); the identification and adoption of effective methods for monitoring adherence to mandated components of interventions; and a more detailed description of credentialing processes, ensuring their inclusion in trial protocols. Delivering these recommendations will strengthen the rigor of future pancreatic surgery RCTs, which may, in turn, enhance implementation across clinical practice.

## Supplementary Material

zraf082_Supplementary_Data

## Data Availability

Data are available upon reasonable request.
