# Supplementary material for: Quality assurance of surgical interventions for pancreatic cancer: systematic review of multicentre randomized clinical trials
Source: BJS Open. 2025 Aug 14;9(4):zraf082. doi: 10.1093/bjsopen/zraf082 (PMC12351452; doi:10.1093/bjsopen/zraf082)
Supplement: zraf082_Supplementary_Data [file zraf082_supplementary_data.docx]

**Quality assurance of surgical interventions for pancreatic cancer: systematic review of multicentre randomised controlled trials**

Jack A Helliwell^1,2^, Sophie Rozwadowski^3^, Jing Yi Kwan^2^, Melissa Bautista^2^, Shailesh Shrikhande^4^, Deborah Stocken ^1,5^, Natalie S Blencowe^1,6^, Andrew M Smith^2^, Samir Pathak^2,5^

1. Leeds Institute of Medical Research at St. James’s, University of Leeds, Leeds, UK
2. Department of Pancreatic Surgery, Leeds Teaching Hospitals NHS Trust, Leeds, UK
3. Department of General Surgery, Barnsley Hospitals NHS Foundation Trust, Barnsley, UK
4. Department of Cancer Surgery, Tata Memorial Centre, Mumbai, India
5. Clinical Trials Research Unit, Leeds Institute of Clinical Trials Research, University of Leeds, Leeds, UK
6. Bristol Centre for Surgical Research, Bristol Medical School, Canynge Hall, Whatley Road, Bristol, UK

**Address Correspondence to:** Jack A Helliwell; Room 7.19, Clinical Sciences Building, Leeds Institute of Medical Research at St. James’s, University of Leeds, LS9 7TF Email: [jackhelliwell@doctors.org.uk](mailto:jackhelliwell@doctors.org.uk)

**Supplementary Materials - Index**

| **Supplementary Methods** |  |
| --- | --- |
| Supplementary Table 1. Search strategy | *page 1* |
| **Supplementary Results** |  |
| Supplementary Table 2. Study characteristics of pancreatoduodenectomy RCTs | *page 2* |
| Supplementary Table 3. Study characteristics of left pancreatectomy RCTs  Supplementary Table 4. Descriptions of surgical interventions in pancreatic surgery trials  Supplementary Table 5. Standardisation of surgical interventions in pancreatic surgery trials  Supplementary Table 6. Methods of measuring intervention adherence in pancreatic surgery trials  Supplementary Table 7. Methods of defining surgeon and/or unit entry criteria in pancreatic surgery trials | *page 5*  *page 7*  *page 11*  *page 14*  *page 16* |
|  |  |

Supplementary Table 1. Search strategy

| 1 | Pancreas |
| --- | --- |
| 2 | Pancreatic |
| 3 | 1 OR 2 |
| 4 | Surgery |
| 5 | Operative surgical procedures |
| 6 | Pancreaticoduodenectomy |
| 7 | Pancreatoduodenectomy |
| 8 | Pancreatic head resection |
| 9 | Pancreatectomy |
| 10 | Pancreatic resection |
| 11 | Duodenum-preserving pancreatic head resection |
| 12 | Duodenopancreatectomy |
| 13 | Pylorus-preserving pancreaticoduodenectomy |
| 14 | DPPHR |
| 15 | PPPD |
| 16 | Pancreatic enucleation |
| 17 | Whipple surgery |
| 18 | Whipple procedure |
| 19 | 4 OR 5 OR 6 OR 7 OR 8 OR 9 OR 10 OR 11 OR 12 OR 13 OR 14 OR 15 OR 16 OR 17 OR 18 |
| 20 | 3 AND 19 |
| 21 | [Limit to year = 2000 to Current] |
| 22 | [Limit to Randomised Controlled Trial] |
| 23 | [Limit to English language] |
| 24 | [Remove duplicates] |
|  | = 2374 |

Supplementary Table 2. Study characteristics of pancreat~~ic~~oduodenectomy RCTs

| **Study**  **(Year)** | **Country** | **Journal** | **Intervention** | **Comparator** | **Primary outcome** | **Number of centres** | **Number of Surgeons** | **Number of participants** |
| --- | --- | --- | --- | --- | --- | --- | --- | --- |
| Wang *et al.* (2023) ^7^ | China | Journal of Advanced Research | Extended lymphadenectomy | Standard lymphadenectomy | 3-year overall survival | 3 | NA | 170 |
| Lin *et al.*  (2023) ^8^ | China | Cancer Communications | Extended resection (retroperitoneal nerve resection) | Standard resection | Overall survival | 6 | NA | 468 |
| Yamada *et al.* (2020) ^9^ | Japan | Annals of Gastroenterological Surgery | Right half dissection of SMA nerve plexus | Total preservation of nerve plexus | Local recurrence | 3 | NA | 74 |
| Welsch *et al.* (2022) ^10^ | Germany | British Journal of Surgery | Falciform ligament wrap around hepatic/GDA | No falciform ligament wrap | Post-pancreatectomy haemorrhage | 8 | NA | 417 |
| Toyama *et al.* (2021) ^11^ | Japan | Annals of Surgery | Retrocolic alimentary tract reconstruction | Antecolic reconstruction | Delayed gastric emptying | 9 | NA | 212 |
| Wang *et al.* (2021) ^12^ | China | The Lancet Gastroenterology & Hepatology | Laparoscopic approach | Open approach | Length of hospital stay | 14 | 14 | 656 |
| Sabater *et al.* (2019) ^13^ | Spain | Annals of Surgery | Artery-first approach | Standard approach | R0 resection rate | 10 | NA | 153 |
| van Hilst *et al.* (2019) ^14^ | the Netherlands | The Lancet Gastroenterology & Hepatology | Laparoscopic approach | Open approach | Time to functional recovery | 4 | NA | 99 |
| Schindl *et al.*  (2018) ^15^ | Austria | British Journal of Surgery | Pancreatojejunostomy sealed with fibrin patch | Pancreatojejunostomy with no fibrin patch | Post-operative pancreatic fistula | 6 | NA | 142 |
| Witzigmann *et al.* (2016) ^16^ | Germany | Annals of Surgery | No routine intra-abdominal drain | Intra-abdominal drain | Need for invasive re-intervention | 2 | NA | 438 |
| Jang *et al.*  (2016) ^17^ | South Korea | British Journal of Surgery | External pancreatic stenting of pancreaticojejunostomy | Internal pancreatic stenting of pancreaticojejunostomy | Post-operative pancreatic fistula | 4 | 6 | 328 |
| Sakamoto *et al.* (2016) ^18^ | Japan | Journal of Gastrointestinal Surgery | Hand-sewn duodenojejunostomy | Circular stapled duodenojejunostomy | Delayed gastric emptying | 2 | NA | 95 |
| Keck *et al.*  (2016) ^19^ | Germany | Annals of Surgery | Pancreatogastrostomy | Pancreatojejunostomy | Post-operative pancreatic fistula | 14 | NA | 320 |
| Van Buren *et al.* (2014) ^20^ | USA | Annals of Surgery | Intra-peritoneal drain placement | No intraperitoneal drain placement | Grade II or greater complication rate | 9 | 15 | 137 |
| Jang *et al.*  (2014) ^21^ | Japan | Annals of Surgery | Extended resection | Standard resection | Overall survival | 7 | 17 | 169 |
| Figueras *et al.* (2013) ^22^ | Spain | British Journal of Surgery | Pancreaticojejunostomy | Invaginated pancreaticogastrostomy | Post-operative pancreatic fistula | 2 | NA | 123 |
| Topal *et al.*  (2013) ^23^ | Belgium | Lancet Oncology | Pancreaticojejunostomy | Pancreaticogastrostomy | Post-operative pancreatic fistula | 8 | NA | 329 |
| Ke *et al.*  (2013) ^24^ | China | Surgery | Roux-en-Y reconstruction with isolated pancreatic drainage | Conventional loop drainage | Post-operative pancreatic fistula | 6 | NA | 216 |
| Uzunoglu *et al.* (2012) ^25^ | Germany | Annals of Surgery | Ultrasonic dissection | Conventional dissection | Operation time | 3 | NA | 101 |
| Nimura *et al.* (2012) ^26^ | Japan | Journal of Hepato-Biliary-Pancreatic Sciences | Extended lymphadenectomy | Standard lymphadenectomy | Long-term survival | 14 | NA | 101 |
| Pessaux *et al.* (2011) ^27^ | France | Annals of Surgery | External pancreatic duct stent | No pancreatic duct stent | Post-operative pancreatic fistula | NA | NA | 158 |
| Berger *et al.*  (2009) ^28^ | USA | Journal of the American College of Surgeons | Duct-to-mucosa pancreaticojejunostomy | Invaginated pancreaticojejunostomy | Post-operative pancreatic fistula | 2 | 8 | 197 |
| Duffas *et al.*  (2005) ^29^ | USA | The American Journal of Surgery | Pancreaticogastrostomy | Pancreaticojejunostomy | Post-operative intra-abdominal complications | 14 | NA | 149 |
| Tran *et al.*  (2004) ^30^ | the Netherlands | Annals of Surgery | Pylorus-preserving pancreaticoduodenectomy | Standard Whipple | Blood loss, operation time, hospital stay | 7 | NA | 170 |
| Suc *et al.*  (2003) ^31^ | France | Annals of Surgery | Occlusion of main pancreatic duct with fibrin glue | No use of fibrin glue | Intra-abdominal complications | 15 | NA | 182 |
| Tran *et al.*  (2002) ^32^ | the Netherlands | Annals of Surgery | Occlusion of main pancreatic duct with Ethibloc, Neoprene or Tissucol | Pancreaticojejunostomy | Post-operative complications | 2 | NA | 169 |
| Takano *et al.*  (2000) ^33^ | Japan | British Journal of Surgery | Pancreaticogastrostomy | Pancreaticojejunostomy | Post-operative complications | 2 | NA | 142 |

NA = not available.

Supplementary Table 3. Study characteristics of left pancreatectomy RCTs

| **Study**  **(Year)** | **Country** | **Journal** | **Intervention** | **Comparator** | **Primary outcome** | **Number of centres** | **Number of Surgeons** | **Number of participants** |
| --- | --- | --- | --- | --- | --- | --- | --- | --- |
| Korrel *et al.*  (2023) ^34^ | the Netherlands | The Lancet Regional Health Europe | Minimally invasive approach | Open approach | Radical resection (R0) | 35 | NA | 261 |
| Merdrignac *et al.* (2022) ^35^ | France | Annals of Surgery | Polyglycolic acid reinforced stapled transection of pancreas | Standard stapled transection of pancreas | Post-operative pancreatic fistula | 7 | NA | 199 |
| Uranues *et al.* (2021) ^36^ | Austria | Annals of Surgery Open | Hemopatch reinforced pancreatic stump | No hemopatch reinforcement | Post-operative pancreatic fistula | 17 | NA | 315 |
| Yamada *et al.* (2021) ^37^ | Japan | JAMA Surgery | Combined division of splenic vein and pancreas | Separate division of splenic vein | Post-operative pancreatic fistula | 45 | NA | 316 |
| Landoni *et al.* (2022) ^38^ | Italy | Surgical Endoscopy | Ultrasonic transection of pancreas | Stapled transection | Post-operative pancreatic fistula | 2 | NA | 145 |
| Wennerblom *et al.* (2021) ^39^ | USA | British Journal of Surgery | Extracellular matrix reinforced stapled transection of pancreas | Standard stapled transection of pancreas | Post-operative pancreatic fistula | 4 | NA | 106 |
| Kondo *et al.*  (2019) ^40^ | Japan | Society of Surgical Oncology | Polyglycolic acid reinforced stapled transection of pancreas | Standard stapled transection of pancreas | Post-operative pancreatic fistula | 9 | NA | 120 |
| de Rooij *et al.* (2019) ^41^ | the Netherlands | Annals of Surgery | Minimally invasive approach | Open approach | Time to functional recovery | 14 | NA | 108 |
| Van Buren *et al.* (2017) ^42^ | USA | Annals of Surgery | Placement of intraperitoneal drain | No intraperitoneal drain | Grade 2 or higher complication rate | 14 | NA | 344 |
| Uemura *et al.* (2017) ^43^ | Japan | British Journal of Surgery | Duct-to-mucosa pancreaticogastrostomy of the pancreatic stump | Hand-sewn closure of pancreatic stump | Post-operative pancreatic fistula | 3 | NA | 73 |
| Jang *et al.*  (2017) ^44^ | South Korea | JAMA Surgery | Wrapping of polyglycolic acid mesh around pancreatic stump | No polyglycolic acid mesh | Post-operative pancreatic fistula | 5 | NA | 97 |
| Shubert *et al.* (2016) ^45^ | USA | Journal of Surgical Research | TissueLink applied to pancreatic stump | SEAMGUARD applied to pancreatic stump | Post-operative pancreatic fistula | 2 | NA | 67 |
| Park *et al.*  (2016) ^46^ | South Korea | Japanese Society of Hepato-Biliar-Pancreatic surgery | Wrapping of pancreatic stump with TachoSil patch | No wrapping wit patch | Post-operative pancreatic fistula | 5 | NA | 101 |
| Kawal *et al.*  (2016) ^47^ | Japan | Annals of Surgery | Pancreaticojejunostoy of pancreatic stump | Stapled closure of pancreatic stump | Post-operative pancreatic fistula | 5 | NA | 123 |
| Cunha *et al.*  (2015) ^48^ | France | American Journal of Surgery | Tachosil sponge on the pancreatic stump | No Tachosil on the pancreatic stump | Post-operative pancreatic fistula | 45 | NA | 270 |
| Carter *et al.*  (2013) ^49^ | USA | Journal of Gastrointestinal Surgery | Stapled/ sutured closure of pancreatic stump plus falciform ligamnent patch and fibrin glue | Stapled/ sutured closure of pancreatic stump only | Post-operative pancreatic fistula | 2 | NA | 109 |
| Montorsi *et al.* (2012) ^50^ | Italy | Annals of Surgery | Tachosil patch applied to pancreatic stump | No Tachosil patch | Post-operative pancreatic fistula | 19 | NA | 275 |
| Diener *et al.*  (2011) ^51^ | Germany | The Lancet | Stapled closure of pancreatic stump | Hand-sewn closure of pancreatic stump | Combination of post-operative pancreatic fistula and death | 21 | NA | 352 |

NA = Not available.

Supplementary Table 4. Descriptions of surgical interventions in pancreatic surgery trials

| **Study (Year)** | **Intervention** | **Description (verbatim)** |
| --- | --- | --- |
| Wang *et al.* (2023) | Laparoscopic approach | *“The techniques used for LPD and OPD have been previously described. The LPD group only included patients who intended to undergo total laparoscopic surgery. Patients who intended to undergo laparoscopic-assisted surgery were excluded.”* |
| Lin *et al.* (2023) | Extended resection | *“During EPD, the following nerve tissues at the retroperitoneum and LNs around the pancreatic head were dissected: (I) nerves and soft tissues between the inferior vena cava (including the aortic plexus) and abdominal aorta (including LN stations 16a2 and 16b1);… and (VI) the nerves and soft tissues in the dense postpancreatic connective tissues that fixed the pancreas at the celiac trunk-aorta-SMA artery axis.”* |
| Korrel *et al.* (2023) | Minimally invasive approach | *“Surgical procedures were performed according to the left radical pancreatectomy principles, rather than the distal pancreatectomy principles.”* |
| Yamada *et al.* (2020) | Right half dissection of SMA nerve plexus | *“The nerve plexus was dissected from the bifurcation of the middle colic artery to the root of the SMA.”* |
| Welsch *et al.* (2022) | Falciform ligament wrap | *“Patients in the intervention group underwent pancreatoduodenectomy with intraoperative coverage of the hepatic artery including the gastroduodenal artery stump using the pedicled falciform ligament wrap in a standardized fashion.”* |
| Toyama *et al.* (2021) | Retrocolic alimentary tract reconstruction | *“Retrocolic alimentary tract reconstruction was performed using a method similar to "vertical retrocolic gastroorduodenojejunostomy… During antecolic reconstruction, the anastomosis was positioned anterior to the transverse colon”* |
| Wang *et al.* (2021) | Laparoscopic approach | *“The laparoscopic pancreaticoduodenectomy group only included patients who were intended for total laparoscopic surgery; laparoscopic assisted or robotic-assisted cases were excluded”* |
| Sabater *et al.* (2019) | Artery-first approach | *“‘Artery-first approach’’ pancreatoduodenectomy (AFA-PD) is characterized by early evaluation of involvement of the main arterial vasculature before irreversible surgical steps are performed as well as meticulous dissection of arterial planes and clearance of retropancreatic tissue.”* |
| Van Hilst *et al.* (2019) | Laparoscopic approach | *“In total, four to five (2 x 12 mm and 2 x 5 mm to 3 x 5 mm) trocars are placed in a semi-circular fashion centred around the supraumbilical camara”* |
| Schindl et al. (2018) | Pancreatojejunostomy sealed with fibrin patch | *“the pancreatojejunostomy was sealed with two 9·5 × 4·8‐cm patches of fibrin‐coated collagen (TachoSil®; Takeda Austria, Linz, Austria) that were placed on the anterior and posterior aspect of the anastomosis”* |
| Witzigmann *et al.* (2016) | No routine intra-abdominal drain | *“In the control group 2 passive drains (Easyflow, Robinson, or Jackson-Pratt) were placed in the region of the pancreaticojejunal anastomosis after resection… In the experimental group no drains were placed.”* |
| Jang *et al.* (2016) | External pancreatic stenting of pancreaticojejunostomy | *“A 4–10-Fr Silastic® (Dow Corning, Midland, Michigan, USA)–polyethylene tube was inserted externally or internally into the pancreatic duct as a stent, according to random allocation.”* |
| Sakamoto *et al.* (2016) | Hand-sewn duodenojejunostomy | *“we conducted the present dual-institution RCT to compare the incidence of postoperative DGE after duodenojejunostomy using a circular stapler (CS) with that after conventional HS anastomosis in PpPD”* |
| Jang *et al.* (2014) | Extended resection | *“During extended resection, lymph nodes around the common hepatic artery (LN 8), celiac axis (LN 9), peripancreatic area (LN 13, 17), hepatoduodenal ligament (LN 12), SMA (LN 14), and para-aortic area (LN 16) between the celiac axis and the inferior mesenteric artery were dissected. All soft tissues around the hepatoduodenal ligament were completely dissected and skeletonized. The nerve plexus or ganglion on the right side of the celiac axis and SMA was dissected semicircumferentially”* |
| Figueras *et al.* (2013) | Pancreaticojejunostomy | *“PG was carried out according to the method described by Delcore and co-workers”* |
| Topal *et al.* (2013) | Pancreaticojejunostomy | *“The pancreatic anastomosis was done in an end-to-side telescoped fashion either into the jejunum...The gastro-jejunostomy was done antecolic and no omega-loop was used.”* |
| Ke *et al.* (2013) | Roux-en-Y reconstruction with isolated pancreatic drainage | *“In the RYR group, reconstruction was begun using the transected jejunum and led through the mesocolon, which was anastomosed in an end-to-side fashion to the pancreatic remnant. A separate Roux loop was fashioned for the hep-aticojejunal anastomosis by dividing the jejunum about 60 cm beyond the pancreatic anastomosis. This was anastomosed to the hepatic duct in an end-to-side fashion. The gastrojejunal anastomosis was performed 30 cm downstream from the hepaticojejunostomy. The pancreaticojejunal loop was then sutured to the main loop. The pancreaticojejunal loop was reimplanted 20 cm downstream on the efferent limb of the gastrojejunostomy.”* |
| Nimura *et al.* (2012) | Extended lymphadenectomy | *“For the extended operation, nodes around the common hepatic artery (CHA) (Nos. 8a, 8p), celiac artery (CA) (No. 9), superior mesenteric artery (SMA) (Nos. 14p, 14d) and abdominal aorta (AA) between the origin of the CA and the inferior mesenteric artery (IMA) (Nos. 16a2, 16b1) were uniformly dissected and the hepatoduodenal ligament was skeletonized to remove nodes of Nos. 12a, 12b and 12p.”* |
| Pessaux *et al.* (2011) | External pancreatic duct stent | *“In the stented group, depending on the size of the pancreatic duct, a Fr 3 to 6 polyvinyl catheter with multiple side-holes was inserted into the pancreatic duct.”* |
| Berger *et al.* (2009) | Duct-to-mucosa pancreaticojejunostomy | *“All patients were stratified by pancreatic texture and randomized to either an invagination or a duct to mucosa pancreaticojejunal anastomosis … two widely used methods to accomplish an end to side pancreaticojejunostomy (PJ) after PD: invagination PJ (or “dunking” the pancreatic remnant into the jejunum) or duct to mucosa PJ”* |
| Tran *et al.* (2004) | Pylorus-preserving pancreaticoduodenectomy | *‘The standard, pylorus-preserving resection involved division of the duodenum 2 cm distal to the pylorus with resection of all of the duodenum distal to the transection site, removal of the gallbladder and common bile duct (proximal to the level of the cystic duct junction), resection of the head, neck, and uncinate process of the pancreas (underneath the superior mesenteric vein, lateral from the mesenteric-portal vein axis, flush with the superior mesenteric artery) and removal of the periampullary tumor.”* |
| Suc *et al.* (2003) | Occlusion of main pancreatic duct with fibrin glue | *“Three to 5 mL of rapidly acting fibrin glue (Tissucol) containing 500 IU thrombin (Immuno France) was used to occlude the main pancreatic duct.”* |
| Tran *et al.* (2002) | Occlusion of main pancreatic duct with Ethibond, Neoprene or Tissucol | *“Occlusion of the pancreatic duct was obtained by injection of Ethibloc (n = 18), Neoprene (n = 45), or Tissucol (n = 23) in combination with aprotinin (Trasylol).”* |
| Takano *et al.* (2000) | Pancreaticogastrostomy | *“An invagination anastomosis was constructed with two layers of interrupted 3/0 absorbable sutures from an anterior gastrotomy and with a pancreatic duct tube exiting through the stomach and abdominal wall.”* |
| Merdrignac *et al.* (2022) | Polyglycolic acid reinforced stapled transection of pancreas | *“Patients were randomly assigned in a 1:1 ratio of DP with standard stapling (SS) of the pancreas using “Endo GIA Articulating X-tra Thick reload with Tri-Staple Technology” (black cartridge) versus DP with polyglycolic acid RS using “Endo GIA Articulating Reinforced Reload with Tri-Staple Technology.”* |
| Uranues *et al.* (2021) | Hemopatch reinforced pancreatic stump | *“The goal of this multicenter double-blinded randomized clinical trial was to evaluate the efficacy, safety, and tolerance of Hemopatch affixed to the pancreatic stump in preventing types B and C POPF (B/C POPF) after DP.. Hemopatch is a thin bovine-derived collagen pad coated with a polyethylene glycol monomer that has both hemostatic and tissue sealing properties that have been used in multiple surgical specialties.”* |
| Yamada *et al.* (2021) | Combined division of splenic vein and pancreas | *“The aim of the study was to establish noninferiority in terms of safety of dividing the splenic vein with the pancreatic parenchyma compared with that of the conventional technique of isolating the vein from the pancreas before ligation and division”* |
| Landoni *et al.* (2022) | Ultrasonic transection of pancreas | *“Ultrasonic transection was performed using the Harmonic Focus® + Shears (open surgery) or the Harmonic Ace® + Shears (minimally invasive surgery), HARMONIC, Johnson & Johnson Medical, Ethicon, Tokyo, Japan. Ultrasonic technology uses high-frequency mechanical energy that cuts by cavitational fragmentation and simultaneously seals tissues by coaptive coagulation.”* |
| Wennerblom *et al.* (2021) | Extracellular matrix reinforced stapled transection of pancreas | *“In one group the pancreatic transection margin was treated with use of a stapler associated with biological rein-forcement (reinforced stapling); in the other group only a stapler was used (standard stapling)”* |
| Kondo *et al.* (2019) | Polyglycolic acid reinforced stapled transection of pancreas | “Patients assigned to the reinforced stapler group underwent DP using the Endo GIA tri-staple (Covidien, Tokyo, Japan) with reinforcing sheet for pancreatic transection, whereas those assigned to the bare stapler group underwent DP using the Endo GIA tri-staple without reinforcement.” |
| de Rooij *et al.* (2019) | Minimally invasive approach | “Minimally invasive approach using laparoscopic surgery or robot-assisted surgery” |
| Uemura *et al.* (2017) | Duct-to-mucosa pancreaticogastrostomy of the pancreatic stump | “The duct-to-mucosa pancreaticogastrostomy has been described previously^19,20^. In brief, to create a gastric mucosal pocket, an incision was made in the seromuscular layer of the posterior gastric wall equal to the width of the residual pancreas…” |
| Jang *et al.* (2017) | Wrapping of polyglycolic acid mesh around pancreatic stump | “Mesh reinforcement is a method of wrapping the remnant pancreatic stump after distal pancreatectomy. Among the meshes introduced is polyglycolic acid (PGA) (Neoveil; Gunze) mesh, a bioabsorbable recombinant membrane made of a synthetic polymer with a cellulose-like structure.” |
| Shubert *et al.* (2016) | TissueLink applied to pancreatic stump | “In the TissueLink treatment arm, after pancreatic transection, with any method chosen by the operating surgeon, the pancreatic remnant was treated with TissueLink alone.” |
| Park *et al.* (2016) | Wrapping of pancreatic stump with TachoSil patch | “TachoSil® is a topical, absorbable, fibrin sealant patch that consists of a collagen fleece coated with human fibrinogen and thrombin. Patients were randomized 1:1 to undergo either stapled transection of the pancreas alone (Control group) or stapled transection followed by wrapping of the remnant pancreatic stump with a TachoSil patch (TachoSil group).” |
| Kawai *et al.* (2016) | Pancreaticojejunostoy of pancreatic stump | “After resection of the pancreatic parenchyma, PJ end-to-side anastomosis by a Roux-en-Y limb for the pancreatic stump was performed via a retrocolic route with an appropriate length of the first jejunal loop (at least 30 cm).” |
| Cunha *et al.* (2015) | Tachosil sponge on the pancreatic stump | “The Tachosil sponge was placed on the stump without sutures, overlapping the closure line by at least 2.5 cm and held there for at least 3 minutes to ensure that it stuck to the stump” |
| Montorsi *et al.* (2012) | Tachosil patch applied to pancreatic stump | *“Patients underwent standard suturing or stapling, after which Tachosil was applied, or not, as determined by prior randomization. In patients randomized to the TachoSil group, the suture or staple line was covered with the TachoSil patch, which was placed on the pancreatic margin and firmly maintained in place for at least 3 minutes to ensure its adherence to the pancreatic parenchyma.”* |

Supplementary Table 5. Standardisation of surgical interventions in pancreatic surgery trials

| **Study (Year)** | **Intervention** | **Description (verbatim)** |
| --- | --- | --- |
| Lin *et al.* (2023) | Extended resection | *“6 meetings (April 15, May 20, June 24, July 15, August 12 and September 15, 2012) were held to discuss the surgical videos and unify the surgical procedures before patient enrolment”* |
| Korrel *et al.* (2023) | Minimally invasive approach | *“Previously published surgical standards for oncological resection during distal pancreatectomy were followed: radical antegrade modular pancreatosplenectomy for open procedures and radical ‘no-touch’ left pancreatosplenectomy for minimally invasive procedures. Both surgical procedures included standardised pancreatic transection, standardised lymph node dissection, routine splenectomy with resection of Gerota's (i.e., perirenal) fascia with or without left adrenal gland based on the location of the tumour.”* |
| Yamada *et al.* (2020) | Right half dissection of SMA nerve plexus | *“In Group A (dissection) the nerve plexus was dissected from the bifurcation of the middle colic artery to the root of the SMA, along with the pancreatic head. The remnant SMA nerve plexus that was the nearest to the presenting part of of the tumour was pathologiclly evaluated intraoperatively in at least 2 sites. In Group B (preservation), the pancreatoduodenectomy was performed with exposure and preservation of the nerve plexus in its entire circumference. D2 lymph node dissection was conducted in both groups, thus the only difference was the performance of right half nerve plexus dissection.* *A close‐up photograph of the SMA was taken to confirm the accuracy of the procedure.”* |
| Welsch *et al.* (2022) | Falciform ligament wrap | *“A standard closure of the gastroduodenal artery stump was recommended (polypropylene 4/0 and two clips), but other techniques were allowed, After mobilization from the ventral abdominal wall and liver surface, the falciform ligament is loosely tunnelled with its pedicle (the round ligament) through the common hepatic artery. b It is then wrapped once around the gastroduodenal artery stump for coverage. c The falciform ligament wrap is fixed in place using two to three single stitches (for example, PDS 5/0).* |
| Toyama *et al.* (2021) | Retrocolic alimentary tract reconstruction | *“SSPPD with conventional lymph node dissection, was performed as a standard procedure for pancreatic, bile duct, or periampullary cancers. SSPPD involved division of the stomach.”* |
| Sabater *et al.* (2019) | Artery-first approach | *“Before the recruitment of patients, there was a consensus meeting to discuss the technical details of operative procedures for standardization in participating institutions..”* |
| Schindl *et al.*  (2018) | Pancreatojejunostomy sealed with fibrin patch | *“Operative technique, patch placement and drainage were standardized between the participating centres..”* |
| Witzigmann *et al.* (2016) | No routine intra-abdominal drain | *“The pancreatic resections with pancreaticojejunal anastomosis were performed by the surgeons according to local practice…”* |
| Sakamoto *et al.*  (2016) | Hand-sewn duodenojejunostomy | *“Duodenum was cut at 2 cm distal side of the pylorus ring using linear stapler. After completion of pancreaticojejunostomy and hepaticojejunostomy, the antecolic gastrojejunostomy was performed according to the results of randomization. In group HS, the duodenojejunostomy was performed either by Albert- Lembert method, Gambee anastomosis, or layer-to-layer method with or without Braun anastomosis at the discretion of each attending surgeons (Fig. 2)”* |
| Uzunoglu *et al.* (2012) | Ultrasonic dissection | *“All surgeons involved confirmed sound expertise with use of the Harmonic Wave Ultrasonic dissector (Ethicon Endo-Surgery Inc, Summerville, NJ) in open and laparoscopic surgery before the trial. The surgical technique was standardized between participating centers, and a sample video of surgical technique to be used was provided.”* |
| Nimura *et al.* (2012) | Extended lymphadenectomy | *“For the extended operation, nodes around the common hepatic artery (CHA) (Nos. 8a, 8p), celiac artery (CA) (No. 9), superior mesenteric artery (SMA) (Nos. 14p, 14d) and abdominal aorta (AA) between the origin of the CA and the inferior mesenteric artery (IMA) (Nos. 16a2, 16b1) were uniformly dissected and the hepatoduodenal ligament was skeletonized to remove nodes of Nos. 12a, 12b and 12p. Furthermore, nerve dissection was carried out circumferentially around the CHA and SMA, and semicircumferentially on the right lateral aspect of the CA. The above lymph node station nomenclature was defined according to the General Rules for Surgical and Pathological Studies on Cancer of the Pancreas by The Japan Pancreas Society which was also used in the previously reported Farnell's RCT.* |
| Yamada *et al.* (2021) | Combined division of splenic vein and pancreas | *For the patients in arm A, the splenic vein was isolated from the pancreatic parenchyma and dissected after ligation (eFigure 1 in Supplement 2). For those in arm B, the splenic vein was transected concurrently with the pancreatic parenchyma using the aforementioned stapler (eFigure 2 in Supplement 2). To confirm that the surgical procedures were conducted as allocated at the time of central judgment, 2 photographs (before and after pancreatic transection) were taken for all patients.”* |
| de Rooij *et al.* (2019) | Minimally invasive approach | “Technical details of MIDP and ODP, as performed within the trial, were published previously.21 Steps were essentially similar for MIDP and ODP” |
| Uemura *et al.* (2017) | Duct-to-mucosa pancreaticogastrostomy of the pancreatic stump | “The duct-to-mucosa pancreaticogastrostomy has been described previously19,20. In brief, to create a gastric mucosal pocket, an incision was made in the seromuscular layer of the posterior gastric wall equal to the width of the residual pancreas…” |
| Jang *et al.* (2017) | Wrapping of polyglycolic acid mesh around pancreatic stump | “The consensus on surgical procedures was determined during a meeting of the participating surgeons before the study was started. Patients in the PGA group underwent pancreatectomy, and PGA mesh was tightly wrapped around the pancreatic stump where fibrin glue was first applied followed by stitching with peripancreatic soft tissue.” |
| Shubert *et al.* (2016) | TissueLink applied to pancreatic stump | “Bioabsorbable mesh sleeves (SEAMGUARD,W.L Gore, Flagstaff, AZ) specifically manufactured for the chosen staple depth and cartridge length were placed over the stapler before firing. The operating surgeon must pause for 20 s after closure of the stapler before firing the SEAMGUARD reinforced stapler.” |
| Park *et al.* (2016) | Wrapping of pancreatic stump with TachoSil patch | “Consensus on the surgical procedure was obtained after several meetings of the participating surgeons before commencing the study.” |
| Carter *et al.* (2013) | Stapled/ sutured closure of pancreatic stump plus falciform ligamnent patch and fibrin glue | “The falciform patch (usual size approximately 3 cm by 4 cm) was harvested from the falciform ligament, placed over the transected margin, and secured to the pancreatic capsule using either 3-0 silk or 3-0 polyglactin suture placed at minimum at the 12, 3, 6, and 9 o'clock positions. Vitagel (Orthovita; Malvern, PA), prepared according to standard instructions, was then injected in the potential space between the transected pancreas and the falciform patch and deposited around the periphery of the patch (typically a total of 3 to 6 cc of fibrin glue was used)." |
| Montorsi *et al.* (2012) | Tachosil patch applied to pancreatic stump | “In patients randomized to the TachoSil group, the suture or staple line was covered with the TachoSil patch, which was placed on the pancreatic margin and firmly maintained in place for at least 3 minutes to ensure its adherence to the pancreatic parenchyma. In laparoscopic procedures, the patch was rolled and introduced into the abdominal cavity through a 10-mm trocar.” |
| Diener *et al.* (2011) | Stapled closure of the pancreatic stump | “A detailed manual for the surgical procedures was developed and approved by all participating trial sites. During the first investigator meeting before patient recruitment, the trial-specific techniques were discussed and operative training done. Moreover, concomitant treatment was standardised.” |

Supplementary Table 6. Methods of measuring intervention adherence in pancreatic surgery trials

| **Study (Year)** | **Method of measuring intervention adherence** | **Description (verbatim)** |
| --- | --- | --- |
| Wang *et al.* (2023) | Intra-operative photographs | “Surgical quality control was maintained by using mandatory intraoperative photographs or videos that identified specific surgical fields. Approximately 20% of LPD and OPD photographs or videos were randomly selected for evaluation. These photographs and videos were reviewed, and feedback on the operative quality was regularly provided to the investigators” |
| Lin *et al.* (2023) | Intra-operative photographs | “… details of the operative procedure (including photographs of the operation field and a surgeon questionnaire detailing the operative findings)..” |
| Yamada *et al.* (2020) | Intra-operative photographs | “A close-up photograph of the SMA was taken to confirm the accuracy of the procedure.” |
| Toyama *et al.* (2021) | Review of case report forms | “Case report forms received included surgical data (e.g. retro- or antecolic alimentar tract reconstruction, SSPPD/PPPD, operative duration, and volume of intraoperative blood loss), to review adherence to the study protocol by surgeons and to check quality of surgery.” |
| Wang *et al.* (2021) | Intra-operative photographs | “Surgical quality control was maintained by using mandatory intraoperative photographs or videos that identified specific surgical fields.” |
| Van Hilst *et al.* (2019) | Intra-operative videos | “Six experienced international pancreatic surgeons re reviewed and graded videos from the laparoscopic pancreatoduodenectomy cases using a modified Birkmeyer scoring system.” |
| Jang *et al.* (2016) | Surgeon self-declaration | “To check adherence of the surgeons with the protocol, investigator meetings were held and reviews of case report forms were performed every 6 months after establishment of the protocol.” |
| Jang *et al.* (2014) | Intra-operative photographs | “Photographs of surgical fields were taken after resection and uploaded into the central database to verify optimal extent of surgery.” |
| Uzunoglu *et al.* (2012) | Surgeon self-declaration | “All surgeons were asked to confirm sufficient usage of the ultrasonic device in the operation note.” |
| Nimura *et al.* (2012) | Intra-operative photographs, Pathological specimen review | “To assess adherence to the lymphadenectomy protocol, intraoperative pictures had to be taken, and the dissection status of all nodal stations and the number of retrieved lymph nodes were recorded on case report forms by surgeons and pathologists… Also, at the regularly organized meetings of the study group, participating surgeons observed intraoperative photographs of both types of procedures to ensure consistency of extension of lymphadenectomy.” |
| Yamada *et al.* (2021) | Intra-operative photographs | “To confirm that the surgical procedures were conducted as allocated at the time of central judgment, 2 photographs (before and after pancreatic transection) were taken for all patients. Central judgment was conducted biannually for all registered patients. At that time, the photographs were reviewed by 2 members of the committee.” |
| Van Buren *et al.* (2017) | Review of operation notes | “Operative notes, anesthesia records, hospital notes, discharge summaries, and other supporting documents were electronically transferred to the coordinating center and reviewed to validate the information being prospectively entered into the secure, Web-based electronic data capture system (Velos Inc, Fremont, CA). The coordinating center followed quality assurance procedures to assure the timely and accurate completion of all electronic case report forms” |
| Park *et al.* (2016) | Intra-operative photographs | “After randomization, all clinical and pathologic data, including operation field photographs, were stored in a central database.” |
| Diener *et al.* (2011) | Intra-operative photographs | “Photographs for each patient were uploaded via the DISPACT trial homepage to confirm compliance of surgical intervention and were reviewed by the masked surgical review board consisting of three surgeons.” |

Supplementary Table 7. Methods of defining surgeon and/or unit entry criteria in pancreatic surgery trials

| **Study (Year)** | **Required level of surgeon and/or unit expertise** | **Description (verbatim)** |
| --- | --- | --- |
| Lin *et al.* (2023) | Surgeon | “all the surgeons participating in this trial had rich surgical experience (the cumulative number of pancreatoduodenectomies exceeded 300 surgeries)” |
|  | Unit | “all participating hospitals were high‐volume pancreatic surgery centers (completing more than 100 pancreatoduodenectomy operations each year)” |
| Korrel *et al.* (2023) | Surgeon | “… at least 50 MIDPs prior to start of trial enrolment.” |
|  | Unit | “… each participating surgical team had to perform at least 15 distal pancreatectomies (any diagnosis) annually.” |
| Van Hilst *et al.* (2019) | Surgeon | “All participating surgeons had performed 50 or more advanced laparoscopic gastrointestinal procedures, 50 or more pancreatoduodenectomies (either laparoscopic or open), completed the training programme in laparoscopic distal pancreatectomy and the training programme in laparoscopic pancreatoduodenectomy, and had performed 20 or more laparoscopic pancreatoduodenectomies.” |
|  | Unit | “All participating centres performed at least 20 pancreato­duodenectomies annually, of which at least ten were laparoscopic.” |
| De Rooij *et al.* (2019) | Surgeon | “Had performed >50 advanced minimally invasive gastrointestinal procedures (ie, beyond diagnostic laparoscopy, cholecystectomy, and appendectomy), >20 distal pancreatectomies (either MIDP or ODP), and >5 MIDPs. |
|  | Unit | “All participating centers performed at least 20 pancreatoduodenectomies annually according to the nationwide Dutch volume threshold for pancreatic surgery.” |
| Wang *et al.* (2021) | Surgeon | “Eligible surgeons were required to meet the following criteria to be considered experienced: (1) performed at least 104 LPD operations and 104 OPD operations;19 (2) completed the MITG­P­CPAM LPD training programme; and (3) submitted unedited videos of themselves doing LPD and OPD procedures that were considered as adeptly done by independent exper.t.” |
| Jang *et al.* (2016) | Surgeon | “All operations were performed by one of six surgeons specializing in pancreatic surgery, who performed between 30 and 50 PDs per year.” |
| Jang *et al.* (2014) | Surgeon | “All of whom performed more than 30 pancreatoduodenectomies per year.” |
| Topal *et al.* (2013) | Surgeon | “Surgeons with several years of expertise in pancreatico-duodenectomy procedures and who had done a minimum of five pancreaticolfogastrostomy and at least five pancreaticojejunostomy reconstruction procedures were eligible to participate in the study.” |
| Nimura *et al.* (2012) | Surgeon | “Surgeons who had experience of more than 50 cases of extended lymphadenectomy for PD” |
| Landoni *et al.* (2022) | Surgeon | “Had a personal annual caseload exceeding 50 major pancreatic resections and had completed the learning curve for both open and minimally invasive DP.” |
| Toyama *et al.* (2021) | Unit | “Participating institutions that had Board Certified Surgeon/s in Gastroenterology (certified by The Japanese Society of Gastroenterological Surgery) on their staff met the eligibility criterion for this study.” |
| Sabater *et al.* (2019) | Unit | “Centers participating in the study had to have a minimum volume of 20 pancreatic resections per year.” |
| Schindl *et al.* (2018) | Unit | “Each [unit] of which had an annual frequency of more than 20 pancreatic resections” |
| Keck *et al.* (2016) | Unit | “with a median case load of 78 major pancreatic resections per year.” |
| Van Buren *et al.* (2014) | Unit | “9 academic high-volume (∼50 PD/yr) pancreas surgery centers in the United States.” |
| Van Buren *et al.* (2017) | Unit | “50 pancreas resections/year” |
| Jang *et al.* (2017) | Unit | “Each of which performed more than 100 pancreatectomies per year and submitted case report forms without rule violation.” |
| Diener *et al.* (2011) | Unit | “Participating centres had to do at least ten pancreatic resections per year.” |
